# Stress cardiac magnetic resonance vs. fractional flow reserve–guided management for intermediate coronary stenosis: a single-center retrospective propensity-matched study with 12-month follow-up

**DOI:** 10.3389/fcvm.2026.1808223

**Published:** 2026-06-17

**Authors:** Qingru Meng, Xia Zhang, Zhendong Wu, Qianwei Han, Yulong Sun, Wenqi Yu, Ruihong Zhai

**Affiliations:** 1 Department of Cardiology, Mudanjiang Cardiovascular Hospital, Mudanjiang, China; 2 Department of Internal Medicine, Yantai Qishan Hospital, Yantai, China; 3 Department of Cardiology, Xinchang County People’s Hospital, Shaoxing, China

**Keywords:** cardiac magnetic resonance, cost-effectiveness, fractional flow reserve, intermediate coronary stenosis, revascularization strategy

## Abstract

**Purpose:**

Invasive fractional flow reserve (FFR) is currently regarded as a reference standard for the functional assessment of intermediate coronary lesions; however, stress perfusion cardiac magnetic resonance (CMR) offers a non-invasive alternative that potentially streamlines clinical decision-making. Evidence comparing the real-world clinical effectiveness and economic implications of these two modalities, particularly within resource-constrained healthcare environments, remains sparse. This study sought to evaluate whether a CMR-guided strategy could reduce revascularization rates and hospital costs while showing comparable short-term clinical outcomes to an FFR-guided strategy in a real-world matched cohort.

**Methods:**

We conducted a retrospective cohort study at a tertiary cardiovascular center in China, screening patients with angiographically intermediate coronary stenosis (40%–90% diameter stenosis) evaluated between January 2021 and December 2024. To rigorously control for selection bias inherent in observational data, we implemented a 1:1 propensity score matching (PSM) analysis based on extensive baseline covariates. The primary clinical endpoint was a composite of major adverse cardiovascular events (MACE) at 12 months, defined as cardiac death, non-fatal myocardial infarction, or ischemia-driven target vessel revascularization. Secondary endpoints included procedural efficiency, contrast volume consumption, and total hospitalization costs.

**Results:**

In the final propensity-matched cohort of 240 patients, the CMR-guided strategy was associated with a lower PCI utilization rate than the FFR-guided strategy (34.2% vs. 48.3%; odds ratio 0.55; 95% CI, 0.33 to 0.93; *P* = 0.036). The CMR group also had lower total hospitalization costs (mean difference, -¥9,785.3; 95% CI, -¥13,253.4 to -¥6,317.3; *P* < 0.001) and lower iodinated contrast volume. At 12 months, MACE occurred in 10.0% of patients in the FFR group and 10.8% in the CMR group, with no statistically significant between-group difference (HR 1.05; 95% CI, 0.48 to 2.30; log-rank *P* = 0.902). Exploratory subgroup analyses showed no significant interaction across prespecified subgroups.

**Conclusion:**

In this matched retrospective cohort, CMR-guided management was associated with lower PCI utilization and lower hospitalization costs, while 12-month MACE rates were not significantly different from those observed with FFR-guided management. These findings suggest that CMR-guided evaluation may be a useful gatekeeper approach in selected patients, although confirmation in larger prospective studies is required.

## 1 Introduction

The management of angiographically intermediate coronary stenosis remains challenging in contemporary interventional cardiology, particularly when the functional significance of a visually estimated 40% to 90% diameter stenosis is uncertain (1). It is now axiomatically accepted that angiographic appearance alone correlates poorly with the physiological significance of a lesion; nevertheless, the “oculostenotic reflex” frequently drives decision-making in routine practice, leading to the implantation of stents in vessels that do not subtend ischemic myocardium (2, 3). Such discordance between anatomy and physiology exposes patients to the inherent risks of invasive procedures and long-term dual antiplatelet therapy without conferring a survival benefit (4). Consequently, international guidelines have shifted the paradigm away from purely anatomical revascularization, strongly mandating functional interrogation to verify ischemia prior to intervention. The critical clinical imperative, therefore, is to identify a diagnostic strategy that accurately discriminates hemodynamically significant lesions while optimizing resource allocation and minimizing unnecessary invasive exposure.

Invasive fractional flow reserve (FFR) currently stands as the reference standard for this functional discrimination (5). By quantifying the trans-stenotic pressure gradient under hyperemic conditions, FFR-guided revascularization has been proven to improve event-free survival compared with angiography-guided strategies (6, 7). However, its adoption in daily practice is frequently hampered by its invasive nature, the costs associated with pressure wires, and the procedural time required for adenosine administration (8). Conversely, stress perfusion cardiac magnetic resonance (CMR) has emerged as a formidable non-invasive alternative. Unlike other modalities, CMR offers a comprehensive “one-stop” assessment of myocardial ischemia, ventricular function, and scar viability without ionizing radiation (9). By visualizing the functional consequences of stenosis at the tissue level, a CMR-first strategy theoretically serves as a robust “gatekeeper,” potentially filtering out patients who would derive no benefit from invasive catheterization, thereby streamlining the pathway to the catheterization laboratory (9, 10).

While landmark randomized controlled trials, such as MR-INFORM and related physiology-guided revascularization studies, have suggested broadly comparable clinical outcomes between perfusion imaging–based and invasive physiology–based strategies in selected populations, the external validity of these findings in unselected, real-world populations remains under-explored (8, 9). This evidence gap is particularly pronounced within the healthcare landscape of China, where the cost structure diverges significantly from Western models; specifically, the high cost of drug-eluting stents relative to lower labor costs creates a unique economic environment where the “gatekeeper” function of diagnostic imaging could yield disproportionate economic dividends (11). Furthermore, observational comparisons are frequently plagued by indication bias—where sicker patients are preferentially channeled toward invasive assessment—necessitating rigorous statistical adjustment to isolate the true effect of the diagnostic strategy itself (12).

To delineate the comparative effectiveness and economic implications of these two modalities in a real-world setting, we conducted a retrospective cohort study utilizing propensity score matching to mitigate selection bias. We sought to evaluate whether a non-invasive CMR-guided strategy was associated with reduced revascularization rates and lower total hospitalization costs, while comparing 12-month clinical outcomes with those observed under an invasive FFR-guided strategy. This analysis aims to provide pragmatic evidence to inform revascularization decision pathways in resource-constrained healthcare systems.

## 2 Methods

### 2.1 Study design and participants

This single-center, retrospective cohort study was conducted at the Mudanjiang Cardiovascular Hospital, a tertiary cardiovascular referral center in Heilongjiang Province, China. We consecutively screened patients aged 40 to 80 years who presented with stable angina or non-ST-segment elevation acute coronary syndromes (NSTE-ACS) and underwent additional functional assessment for at least one angiographically intermediate native coronary lesion between January 2021 and December 2024. Eligible lesions were those with an estimated diameter stenosis of 40%–90% by visual assessment that, in routine clinical practice, were considered to have uncertain functional significance and were therefore referred for further evaluation with either stress cardiac magnetic resonance or invasive fractional flow reserve. Lesions judged unequivocally severe and referred directly for revascularization without additional functional testing were generally not included. The study protocol complied with the Declaration of Helsinki and was approved by the institutional ethics committee, which waived the requirement for written informed consent because of the retrospective design and anonymized data processing.

Eligibility criteria included the presence of at least one intermediate lesion in a native coronary artery (reference diameter ≥2.5 mm) evaluated by either invasive FFR or stress perfusion Cardiac Magnetic Resonance. To ensure the study population reflected patients potentially eligible for revascularization, we excluded those with ST-segment elevation myocardial infarction within 48 h, prior coronary artery bypass grafting, or chronic total occlusions. Patients with severe hepatic or renal dysfunction (defined as transaminases >3 times the upper limit of normal or creatinine >200 μmol/L), active malignancy, or a life expectancy of less than one year were also excluded to minimize competing risks for mortality. Because of the retrospective design, no prospective sample size calculation was performed. The study sample was therefore determined by the number of consecutive eligible patients available during the prespecified study period.

### 2.2 Procedures and diagnostic strategies

Because of the retrospective design, allocation to stress CMR or invasive FFR was not randomized. In routine clinical practice, the diagnostic modality was selected by the treating heart team according to clinical presentation, timing of invasive angiography, patient suitability for magnetic resonance imaging and adenosine stress, and logistical availability. In general, CMR was preferentially used in clinically stable patients when non-invasive ischemia assessment was feasible, whereas FFR was more commonly used when patients were already in the catheterization laboratory for invasive angiographic evaluation or when CMR was contraindicated or impractical.

Stress CMR was performed on a 3.0-T scanner (GE HDXT, GE Healthcare) using the institutional stress perfusion protocol, which included cine imaging for ventricular function, adenosine stress perfusion imaging, and late gadolinium enhancement (LGE) imaging. A gadolinium-based contrast agent was administered intravenously at 0.1 mmol/kg according to institutional practice. Adenosine was administered intravenously at 140 μg/kg/min. Perfusion assessment in routine practice was based on visual interpretation, and quantitative perfusion mapping was not routinely performed. Inducible ischemia was defined as a reversible perfusion defect involving ≥2 myocardial segments or an ischemic burden ≥10% of the left ventricular mass. When these criteria were not fully concordant, the final interpretation was made by consensus of experienced readers. LGE was used to identify myocardial scar and support interpretation of perfusion findings, but was not used as an isolated indication for revascularization.

Invasive FFR was assessed using a pressure-wire system on a Philips angiography platform. Hyperemia was induced with intravenous adenosine at 140 μg/kg/min. An FFR value ≤0.80 was considered functionally significant, whereas values >0.80 supported deferral of revascularization.

### 2.3 Outcomes and data sources

The primary endpoint was a composite of Major Adverse Cardiovascular Events (MACE) at 12 months, defined as cardiac death, non-fatal myocardial infarction, or ischemia-driven target vessel revascularization. Secondary endpoints included procedural efficiency (PCI utilization rate, stent burden, contrast volume), safety (bleeding events defined as BARC type ≥2), and economic outcomes. Total hospitalization costs were extracted directly from the hospital's electronic billing system and included all expenses related to procedures, ward stay, and medication (reported in RMB). Clinical follow-up data were obtained from electronic medical records and standardized telephone interviews conducted at 6 and 12 months after the index evaluation. Follow-up was considered complete when outcome status at the prespecified time point was available from either source.

### 2.4 Statistical analysis

To mitigate selection bias inherent in observational research, we implemented Propensity Score Matching (PSM). A propensity score for the likelihood of undergoing CMR was estimated using a multivariable logistic regression model incorporating prespecified baseline covariates available before treatment decision-making, including age, sex, BMI, smoking status, hypertension, diabetes mellitus, prior myocardial infarction, left ventricular ejection fraction, and angiographic complexity (SYNTAX score). Patients were matched 1:1 using nearest-neighbor matching without replacement and a caliper width of 0.02. Supplementary balance diagnostics were generated using the same covariates included in the propensity score model. In the matched cohort, continuous variables were compared using Student's t-test or the Wilcoxon rank-sum test, as appropriate, and categorical variables were compared using the *χ*² test or Fisher's exact test. Time-to-event outcomes were analyzed using Kaplan–Meier estimates and compared with the log-rank test. Hazard ratios and 95% confidence intervals were derived from Cox proportional hazards models. A two-sided *P*-value <0.05 was considered statistically significant. All analyses were performed using Python (version 3.8; libraries pandas, lifelines). This study was designed as an exploratory comparative-effectiveness analysis. No formal non-inferiority margin was prespecified, and no formal non-inferiority testing was performed. Therefore, the absence of a statistically significant between-group difference should not be interpreted as proof of non-inferiority or equivalence. Given the limited number of outcome events after matching, analyses of safety outcomes and individual components of the composite endpoint were considered exploratory.

## 3 Results

### 3.1 Study population and propensity matching

**Figure 1:**
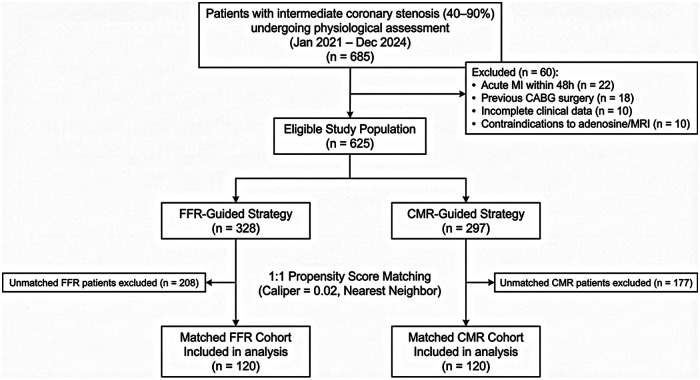
Study flow diagram illustrating patient selection and propensity score matching. The flowchart depicts the screening, enrollment, and matching process for the study cohort. Of 685 consecutive patients with intermediate coronary stenosis assessed between January 2021 and December 2024, 60 were excluded based on prespecified criteria. The remaining 625 eligible patients comprised the unmatched cohort (FFR-guided, *n* = 328; CMR-guided, *n* = 297). After 1:1 propensity score matching with a caliper of 0.02, 120 patients from each group were retained in the final matched analytical cohort. Patients not retained after propensity score matching were not included in the matched analysis. FFR,  fractional flow reserve; CMR,  cardiac magnetic resonance; CABG,  coronary artery bypass grafting; MI, myocardial infarction.

From January 2021 to December 2024, 685 patients with intermediate coronary stenosis were screened for eligibility. After exclusion of 60 patients according to prespecified criteria, 625 patients remained in the unmatched cohort, including 328 in the FFR-guided management group and 297 in the CMR-guided management group (Figure 1). In the unmatched cohort, patients in the FFR group were older and had a higher prevalence of unstable angina, diabetes mellitus, and higher SYNTAX scores than those in the CMR group (Table 1), indicating important baseline imbalances. After 1:1 propensity score matching, 240 patients (120 per group) were included in the final analytical cohort. Matching substantially improved baseline comparability, with all prespecified covariates showing absolute standardized mean differences <0.10 (Table 1; Supplementary Figure S1). Propensity score overlap also improved after matching, although some residual asymmetry remained at the upper tail of the distribution (Supplementary Figure S2).

**Table 1:** Baseline characteristics before and after matching.

Characteristic	Unmatched cohort: FFR group (*n* = 328)	Unmatched cohort: CMR group (*n* = 297)	Unmatched cohort: SMD	Matched cohort: FFR group (n = 120)	Matched cohort: CMR group (n = 120)	Matched cohort: SMD
Demographics						
Age, years	64.5 (9.8)	59.8 (10.2)	0.47	61.5 (10.1)	61.2 (10.4)	0.03
Male sex, no. (%)	223 (68.0)	184 (62.0)	0.13	81 (67.5)	79 (65.8)	0.04
BMI, kg/m²	26.8 (3.6)	26.2 (3.4)	0.17	26.4 (3.5)	26.5 (3.4)	0.03
Clinical Presentation						
Stable Angina, no. (%)	148 (45.1)	205 (69.0)	0.50	70 (58.3)	74 (61.7)	0.07
Unstable Angina, no. (%)	180 (54.9)	92 (31.0)	0.50	50 (41.7)	46 (38.3)	0.07
CCS Class III/IV, no. (%)	115 (35.1)	59 (19.9)	0.35	34 (28.3)	31 (25.8)	0.06
Cardiovascular Risk Factors						
Hypertension, no. (%)	220 (67.1)	169 (56.9)	0.21	78 (65.0)	76 (63.3)	0.04
Diabetes Mellitus, no. (%)	125 (38.1)	71 (23.9)	0.31	41 (34.2)	38 (31.7)	0.05
Dyslipidemia, no. (%)	190 (57.9)	155 (52.2)	0.12	66 (55.0)	68 (56.7)	0.03
Current Smoker, no. (%)	148 (45.1)	135 (45.5)	0.01	54 (45.0)	58 (48.3)	0.07
Previous MI, no. (%)	72 (22.0)	42 (14.1)	0.21	22 (18.3)	19 (15.8)	0.07
Previous PCI, no. (%)	52 (15.9)	30 (10.1)	0.17	14 (11.7)	12 (10.0)	0.05
Family History of CAD, no. (%)	85 (25.9)	74 (24.9)	0.02	30 (25.0)	28 (23.3)	0.04
Laboratory Findings						
LVEF, %	57.2 (8.5)	60.5 (7.2)	0.42	59.1 (8.0)	59.5 (7.8)	0.05
LDL-C, mmol/L	2.9 (0.9)	2.7 (0.8)	0.23	2.8 (0.8)	2.8 (0.9)	0.00
HbA1c, %	7.2 (1.5)	6.5 (1.1)	0.53	6.8 (1.3)	6.7 (1.2)	0.08
eGFR, mL/min/1.73m²	82.5 (18.4)	88.2 (16.5)	0.32	85.1 (17.5)	86.4 (18.0)	0.07
Angiographic Characteristics						
Multivessel Disease, no. (%)	145 (44.2)	95 (32.0)	0.25	48 (40.0)	44 (36.7)	0.07
LAD Proximal Involvement, no. (%)	160 (48.8)	104 (35.0)	0.28	52 (43.3)	50 (41.7)	0.03
SYNTAX Score	16.5 (7.2)	12.8 (5.5)	0.58	14.2 (6.1)	14.0 (5.9)	0.03
Medication on Admission						
Aspirin, no. (%)	315 (96.0)	288 (97.0)	0.05	115 (95.8)	116 (96.7)	0.04
P2Y12 Inhibitor, no. (%)	308 (93.9)	275 (92.6)	0.05	112 (93.3)	110 (91.7)	0.06
Statins, no. (%)	302 (92.1)	270 (90.9)	0.04	108 (90.0)	110 (91.7)	0.06
Beta-blockers, no. (%)	210 (64.0)	160 (53.9)	0.21	70 (58.3)	66 (55.0)	0.07

Data are presented as mean (SD) for continuous variables and number (%) for categorical variables. Propensity score matching was performed using a 1:1 nearest-neighbor algorithm with a caliper of 0.02. BMI, body mass index; CCS, Canadian Cardiovascular Society; CAD, coronary artery disease; MI, myocardial infarction; PCI, percutaneous coronary intervention; LVEF, left ventricular ejection fraction; LDL-C, low-density lipoprotein cholesterol; HbA1c, glycated hemoglobin; eGFR, estimated glomerular filtration rate; LAD, left anterior descending artery; SMD, standardized mean difference. Statistical Note: An SMD > 0.10 is considered to indicate a meaningful imbalance between groups. In the unmatched cohort, significant differences were observed in age, clinical presentation, diabetes prevalence, and angiographic complexity (Syntax score), which were successfully balanced after matching (all SMDs < 0.10).

### 3.2 Procedural characteristics and resource utilization

**Figure 2:**
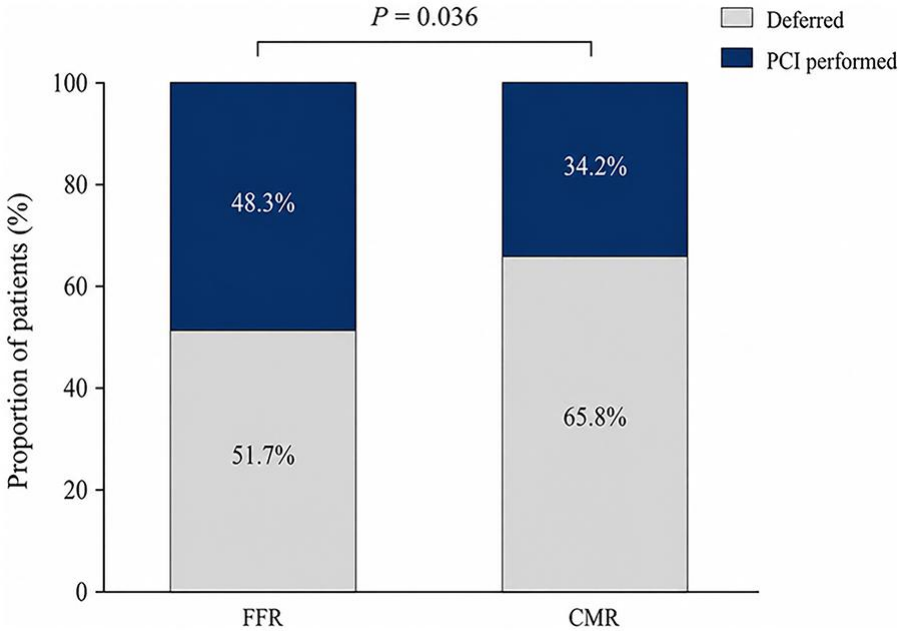
Comparison of revascularization strategies between the FFR-guided and CMR-guided groups. The stacked bar chart illustrates the proportion of patients undergoing percutaneous coronary intervention (PCI, dark blue) versus deferral to medical therapy (light gray) in the matched cohort (*N* = 240). The CMR-guided strategy was associated with a higher deferral rate than the FFR-guided strategy (65.8% vs. 51.7%; *P* = 0.036). FFR, fractional flow reserve; CMR, cardiac magnetic resonance.

In the propensity-matched cohort, the CMR-guided strategy was associated with a lower rate of percutaneous coronary intervention than the FFR-guided strategy (Table 2; Figure 2). PCI was performed in 41 patients in the CMR group and 58 patients in the FFR group, corresponding to PCI rates of 34.2% and 48.3%, respectively (OR 0.55, 95% CI 0.33 to 0.93; *P* = 0.036). Conversely, deferral to medical therapy was more frequent in the CMR group than in the FFR group (65.8% vs. 51.7%; OR 1.80, 95% CI 1.07 to 3.03; *P* = 0.036), indicating that fewer patients proceeded to immediate revascularization after CMR-guided evaluation.

**Table 2:** Procedural characteristics and resource utilization in the propensity-matched cohort.

Variable	FFR group (*n* = 120)	CMR group (*n* = 120)	Effect estimate	*P* value
PCI performed, *n* (%)	58 (48.3)	41 (34.2)	OR 0.55 (95% CI, 0.33 to 0.93)	0.036
Deferred to medical therapy, *n* (%)	62 (51.7)	79 (65.8)	OR 1.80 (95% CI, 1.07 to 3.03)	0.036
Total stents implanted per patient, mean ± SD	1.05 ± 1.27	0.78 ± 1.27	Mean difference −0.27	0.037
Stents per PCI-treated patient, mean ± SD	2.17 ± 0.94	2.27 ± 1.16	Mean difference 0.10	0.876
Contrast volume, mL, mean ± SD	181.6 ± 51.0	98.0 ± 60.7	Mean difference −83.6 (95% CI, −97.8 to −69.3)	<0.001
Total hospital cost, RMB, mean ± SD	49,068.6 ± 11,279.1	39,283.3 ± 15,630.3	Mean difference −9,785.3 (95% CI, −13,253.4 to −6,317.3)	<0.001
Length of stay, days, mean	6.8	5.4	Mean difference −1.4	<0.001

Data are presented as number (%) or mean ± SD, as appropriate. FFR, fractional flow reserve; CMR, cardiac magnetic resonance; PCI, percutaneous coronary intervention; OR, odds ratio; MD, mean difference; CI, confidence interval; RMB, Renminbi.

The overall stent burden per patient was also lower in the CMR group than in the FFR group (0.78 ± 1.27 vs. 1.05 ± 1.27; *P* = 0.037). However, among patients who actually underwent PCI, the number of stents implanted per treated patient was similar between groups (2.27 ± 1.16 vs. 2.17 ± 0.94; *P* = 0.876), suggesting that the difference in total stent burden was mainly driven by the lower proportion of patients proceeding to PCI rather than by fewer stents being used once PCI was performed.

**Figure 3:**
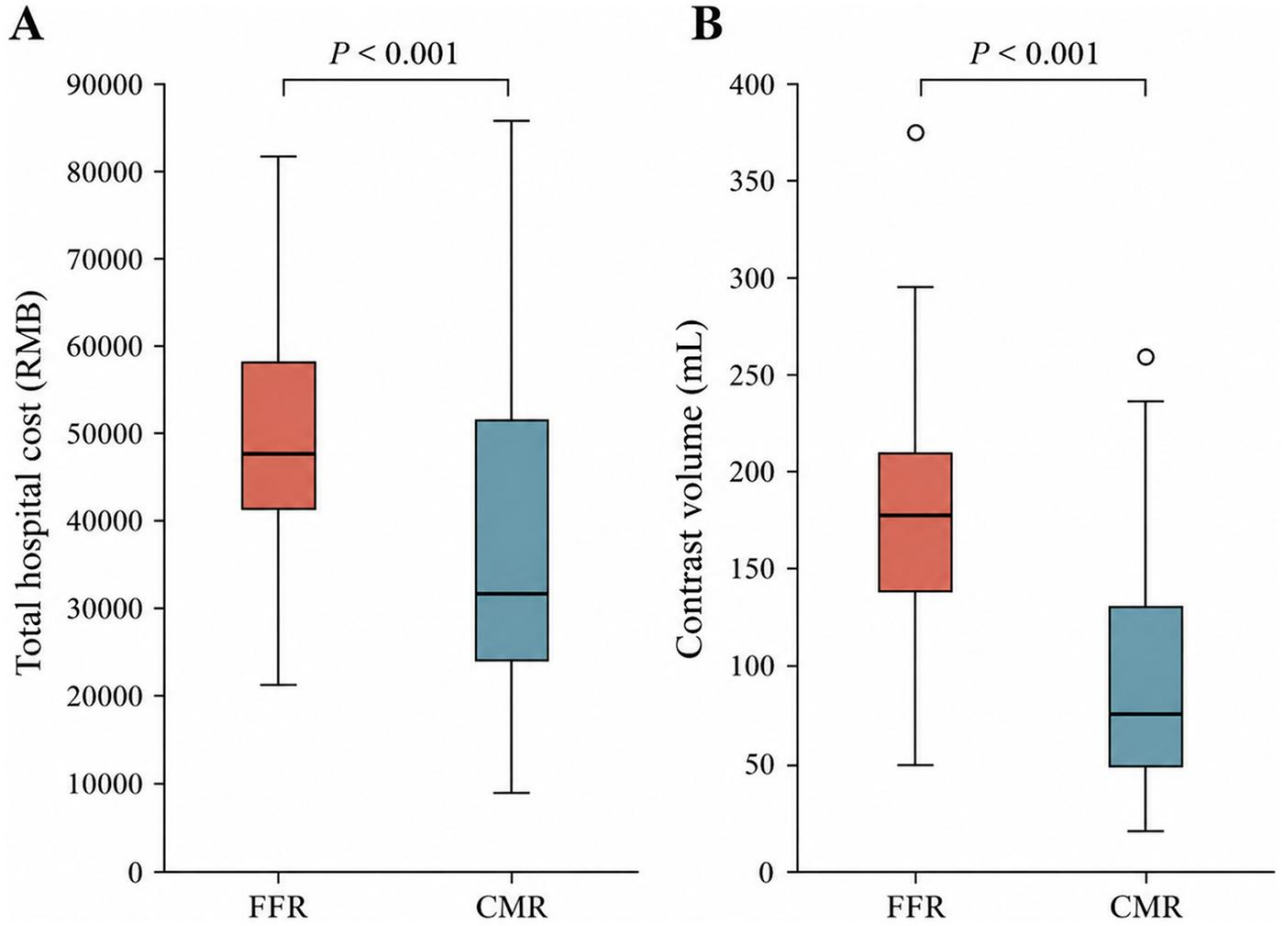
Distribution of economic and procedural resource utilization in the propensity-matched cohort. **(A)** Box plots showing the distribution of total hospitalization costs (in RMB). **(B)** Box plots showing the distribution of iodinated contrast volume (in mL) used during the index hospitalization. Between-group differences were statistically significant for both outcomes (*P* < 0.001). The central line represents the median, the box limits indicate the interquartile range (25th to 75th percentiles), and the whiskers extend to 1.5 times the interquartile range. Outliers are plotted as individual points.

Resource utilization outcomes consistently favored the CMR-guided strategy, as illustrated in Figure 3. The mean iodinated contrast volume was substantially lower in the CMR group than in the FFR group (98.0 ± 60.7 mL vs. 181.6 ± 51.0 mL; mean difference, −83.6 mL; 95% CI, −97.8 to −69.3; *P* < 0.001). Total hospitalization cost was also significantly reduced in the CMR group (RMB 39,283.3 ± 15,630.3 vs. RMB 49,068.6 ± 11,279.1; mean difference, −9,785.3 RMB; 95% CI, −13,253.4 to −6,317.3; *P* < 0.001). For international interpretability, this cost difference corresponds to approximately USD 1,340 using an approximate exchange rate of 1 USD = 7.30 RMB. Thus, compared with FFR-guided management, CMR-guided evaluation was associated with lower PCI utilization, reduced contrast exposure, and lower hospitalization costs in the matched cohort.

### 3.3 Clinical outcomes

**Figure 4:**
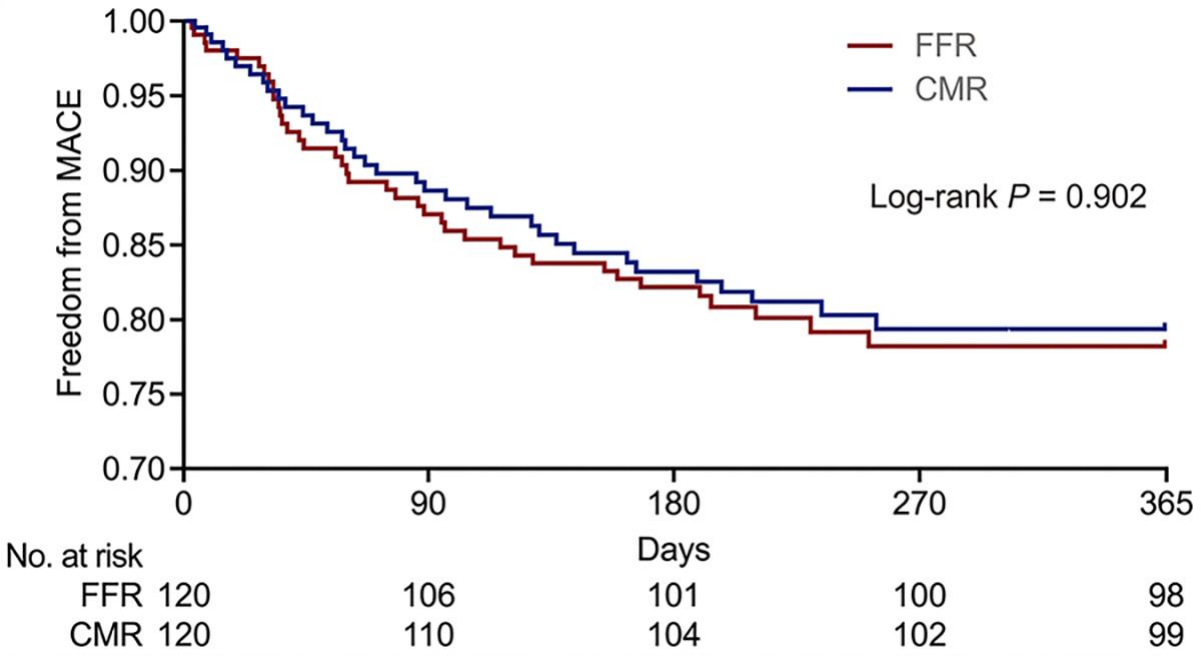
Kaplan–Meier curves for freedom from major adverse cardiovascular events in the propensity-matched cohort. MACE was defined as a composite of cardiac death, non-fatal myocardial infarction, or ischemia-driven target vessel revascularization. There was no statistically significant difference in freedom from MACE between the FFR-guided and CMR-guided strategies at 12 months (log-rank *P* = 0.902). The table below the *x*-axis shows the number of patients at risk at each time point.

Clinical outcomes at 12-month follow-up in the propensity-matched cohort are summarized in Table 3. In the 12-month analysis with administrative censoring at 365 days, MACE occurred in 12 patients in the FFR group and 13 patients in the CMR group, corresponding to crude event rates of 10.0% and 10.8%, respectively. Kaplan–Meier analysis showed no statistically significant difference in freedom from MACE between the two management strategies (log-rank *P* = 0.902) (Figure 4). The Cox proportional hazards model yielded a hazard ratio of 1.05 for CMR vs. FFR (95% CI, 0.48 to 2.30; *P* = 0.903), indicating no significant between-group difference in 12-month MACE in the matched cohort. Bleeding events were numerically more frequent in the CMR group than in the FFR group, but the difference was not statistically significant (8/120 [6.7%] vs. 4/120 [3.3%]; *P* = 0.375). In addition, 12 patients in the matched cohort were lost to follow-up, yielding an overall follow-up completeness of 95.0%. These patients were censored in the time-to-event analysis.

**Table 3:** Clinical outcomes at 12-month follow-up in the propensity-matched cohort.

Clinical outcome	FFR Group (*n* = 120)	CMR Group (*n* = 120)	Effect estimate (95% CI)	*P* value
Primary endpoint				
MACE, No. (%)	12 (10.0)	13 (10.8)	HR 1.05 (0.48–2.30)	0.902
Safety endpoint				
BARC bleeding ≥ type 2, No. (%)	4 (3.3)	8 (6.7)	OR 2.07 (0.61–7.07)	0.375
Follow-up completeness				
Patients lost to follow-up, No. (%)	8 (6.7)	4 (3.3)	OR 0.48 (0.14–1.65)	0.375

Data are presented as number (percentage) of patients. MACE was assessed within 365 days. The hazard ratio for MACE was estimated for CMR versus FFR using a Cox proportional hazards model, and the *P* value was calculated using the log-rank test. Odds ratios for categorical safety and follow-up variables are reported for CMR versus FFR, with *P* values from Fisher's exact test. Abbreviations: BARC, Bleeding Academic Research Consortium; CI, confidence interval; CMR, cardiac magnetic resonance; FFR, fractional flow reserve; HR, hazard ratio; MACE, major adverse cardiovascular events; OR, odds ratio. For MACE, the *P* value shown in the table was derived from the log-rank test; the Cox proportional hazards model yielded HR 1.05 (95% CI, 0.48–2.30; Cox model *P* = 0.903).

Overall, the CMR-guided strategy was not associated with a statistically significant increase in 12-month MACE compared with the FFR-guided strategy. However, because the number of events was limited and the confidence interval was wide, these findings should be interpreted as exploratory rather than as evidence of non-inferiority or clinical equivalence.

### 3.4 Subgroup analysis

**Figure 5:**
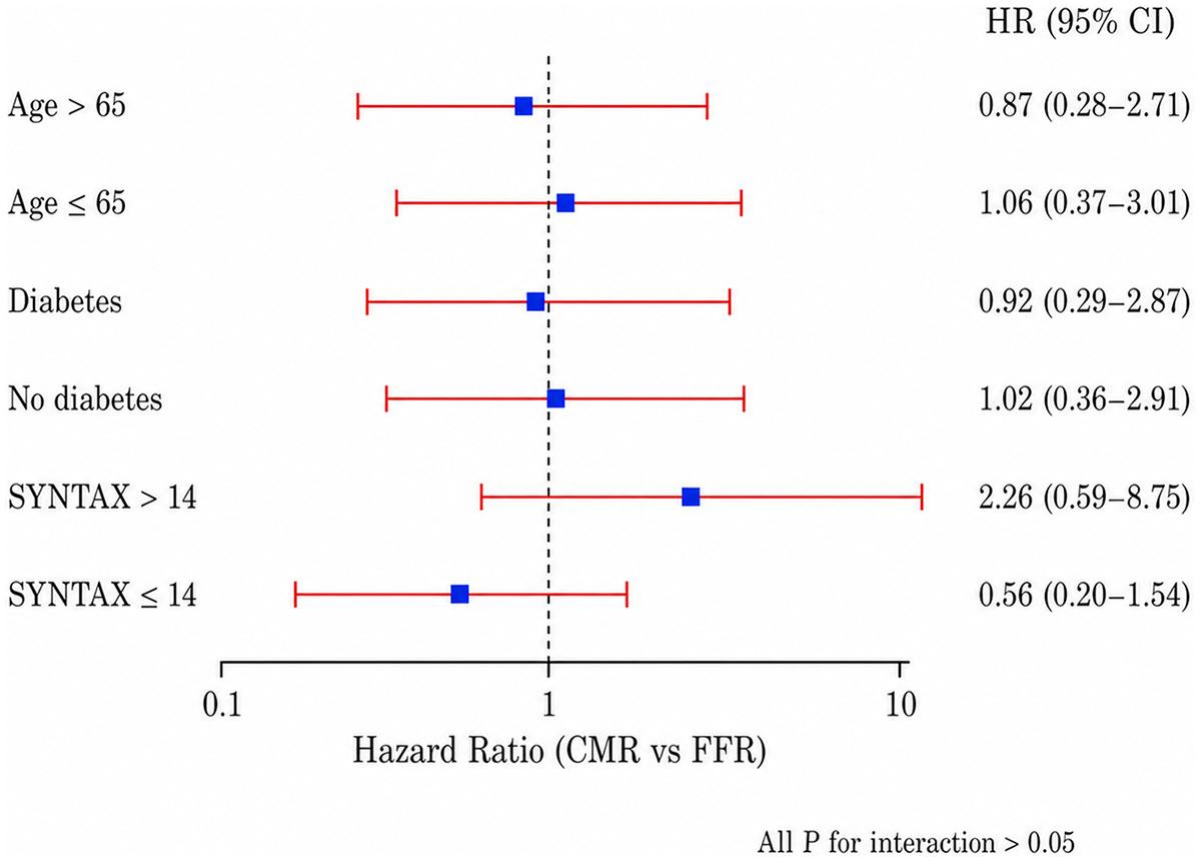
Forest plot of the primary endpoint across prespecified subgroups. The plot displays the hazard ratios (HRs) and 95% confidence intervals (CIs) for MACE (CMR versus FFR) in key clinical subgroups. The vertical line at 1.0 indicates no difference between strategies. The position of the square indicates the point estimate of the HR, and the horizontal line indicates the 95% CI. No significant interactions were observed (all *P* for interaction >0.05), although these subgroup findings should be interpreted cautiously because of the limited number of events.

Exploratory subgroup analyses were performed to assess whether the association between diagnostic strategy and 12-month MACE differed across clinically relevant patient strata. The effect estimates for CMR vs. FFR were generally consistent across age, diabetes status, and SYNTAX score subgroups, with no statistically significant interaction observed in any subgroup comparison (all *P* for interaction >0.05) (Figure 5).

Among patients aged >65 years, the hazard ratio for MACE with CMR vs. FFR was 0.87 (95% CI, 0.28 to 2.71), while the corresponding hazard ratio among patients aged ≤65 years was 1.06 (95% CI, 0.37 to 3.01). Similar findings were observed according to diabetes status, with hazard ratios of 0.92 (95% CI, 0.29 to 2.87) in patients with diabetes and 1.02 (95% CI, 0.36 to 2.91) in those without diabetes. In patients with higher angiographic complexity, defined as SYNTAX score >14, the hazard ratio was 2.26 (95% CI, 0.59 to 8.75), whereas in those with SYNTAX score ≤14, the hazard ratio was 0.56 (95% CI, 0.20 to 1.54).

Overall, no clear evidence of heterogeneity in the association between diagnostic strategy and 12-month MACE was observed across the examined subgroups. However, because the number of events within each subgroup was limited and confidence intervals were wide, these subgroup findings should be interpreted cautiously and considered exploratory.

## 4 Discussion

In this propensity-matched analysis of real-world practice within a tertiary Chinese cardiovascular center, a non-invasive, CMR-guided diagnostic strategy for intermediate coronary stenosis was associated with lower rates of coronary revascularization and reduced total hospitalization costs compared with an invasive FFR-guided strategy. This gain in procedural and economic efficiency was observed alongside similar 12-month MACE rates in the matched cohort, although the study was not designed or powered to establish non-inferiority for clinical safety. These findings suggest that perfusion-based imaging may serve as a potential gatekeeper associated with lower resource use in this local real-world setting.

The observed PCI utilization was lower in the CMR arm than in the FFR arm, decreasing from 48.3% to 34.2%, which was directionally consistent with prior trials evaluating ischemia-guided management strategies, including MR-INFORM (9). This finding may reflect differences between the two physiological paradigms. FFR infers ischemia from trans-stenotic pressure gradients, whereas CMR visualizes the functional consequences of stenosis at the tissue level (7, 10). In clinical practice, the “oculostenotic reflex” often exerts a gravitational pull toward intervention once a catheter is inside the artery; by completing the functional assessment non-invasively, a CMR-first approach effectively decouples the diagnostic workup from the therapeutic procedure, thereby raising the threshold for *ad hoc* stenting (2). Our data suggest that a proportion of anatomically intermediate lesions may not generate sufficient ischemic burden to warrant immediate mechanical revascularization and may be managed with medical therapy in selected patients. The broad angiographic inclusion range should be interpreted in the context of routine clinical care. The 40%–90% threshold represented the range of visually estimated lesions that were referred for additional functional assessment, not a recommendation that every lesion in this range uniformly requires CMR or FFR. In practice, lower-grade lesions were investigated only when functional relevance remained uncertain, whereas lesions judged clearly severe and suitable for direct revascularization were generally not sent for additional testing.

A distinct contribution of this study is its evaluation of economic outcomes within the specific context of the Chinese healthcare landscape. Unlike Western models where labor costs often drive expenditures, medical costs in China are heavily skewed toward high-value consumables (11). Consequently, the avoidance of implantation of drug-eluting stents and the associated reduction in contrast volume translate into disproportionate fiscal savings (11). In our cohort, the CMR-guided strategy yielded a net saving of nearly ¥10,000 per patient. As China transitions toward Diagnosis-Related Group (DRG) payment systems, such efficiency gains are strategically vital (13, 14). In this local context, CMR-guided evaluation may help identify patients who are less likely to require immediate revascularization and may therefore contribute to more efficient resource allocation (5). These economic findings should be interpreted in the context of local hospital pricing and reimbursement structures and may not be directly generalizable to other healthcare systems.

Regarding safety, the Kaplan–Meier curves and event rates showed no statistically significant difference in 12-month MACE between the two groups; however, these observational findings should not be interpreted as formal proof of non-inferiority or equivalence (15). The lower intervention rate in the CMR group was not accompanied by a statistically significant increase in short-term MACE, but the limited number of events precludes definitive safety conclusions (4).

These findings should be interpreted in light of several limitations. First, although propensity score matching improved baseline comparability, the matched cohort remained modest in size (120 patients per group), with only 12 primary endpoint events in the FFR group and 13 in the CMR group. The study was therefore likely underpowered for safety comparisons, particularly for the individual components of the composite endpoint, as reflected by the wide confidence intervals around several effect estimates. Accordingly, the absence of statistically significant between-group differences should not be interpreted as proof of non-inferiority, equivalence, or definitive safety comparability. In addition, 12 patients in the matched cohort were lost to follow-up, yielding an overall follow-up completeness of 95.0%. Although these patients were censored in the time-to-event analysis, loss to follow-up may still have affected event-rate estimates. Second, this was a retrospective, single-center study, and the choice between stress CMR and invasive FFR was made in routine clinical practice rather than by random allocation. Although we expanded the description of the institutional selection process and used propensity score matching to reduce measured confounding, residual confounding and treatment-selection bias from unmeasured factors may still persist, including operator preference, test availability, scheduling considerations, perceived image quality, subtle differences in comorbidity burden, MRI-related contraindications, socioeconomic status, and patient preference (16). Therefore, the observed between-group differences should be interpreted as associations within a real-world workflow rather than as causal effects of the diagnostic modality itself. Detailed lesion-level stenosis strata and the lesion-level relationship between visual stenosis severity, functional test results, and subsequent management decisions were not consistently available in this retrospective dataset; therefore, the present study should be interpreted as a patient-level management-strategy comparison rather than a lesion-level diagnostic accuracy study. Third, as a single-center study, the cost analysis reflects local pricing structures and practice patterns, which may limit generalizability to other regions with different reimbursement models (17). Finally, although 12-month follow-up captures early clinical events, longer-term follow-up is needed to assess the durability of these findings (18). Overall, the present results should be regarded as findings from a real-world retrospective comparative-effectiveness analysis rather than as definitive evidence of the superiority of one modality over the other.

## 5 Conclusion

In this propensity-matched cohort, CMR-guided evaluation for intermediate coronary stenosis was associated with lower PCI utilization and lower hospitalization costs, while 12-month MACE rates were not significantly different from those observed with FFR-guided management. These findings suggest that CMR-guided evaluation may be a useful gatekeeper approach in selected patients, but confirmation in larger prospective studies is required.

## Data Availability

The raw data supporting the conclusions of this article will be made available by the authors, without undue reservation.

## Supplementary material

Supplementary Figure S1 Covariate balance before and after propensity score matching. The Love plot displays the absolute standardized mean differences for the prespecified covariates included in the propensity score model: age, sex, BMI, smoking status, hypertension, diabetes mellitus, prior myocardial infarction, left ventricular ejection fraction, and SYNTAX score. Red circles indicate the unmatched cohort and blue circles indicate the matched cohort. The dashed vertical line marks an absolute standardized mean difference of 0.10. After matching, all included covariates showed an absolute standardized mean difference <0.10.

Supplementary Figure S2 Propensity score distributions before and after matching. **(A)** Distribution before matching. **(B)** Distribution after matching. The upper bars represent the FFR group, and the lower mirrored bars represent the CMR group. Propensity score overlap improved after matching, although some residual asymmetry remained at the upper tail of the distribution.
