# Supplementary figures and images for: Stress cardiac magnetic resonance vs. fractional flow reserve–guided management for intermediate coronary stenosis: a single-center retrospective propensity-matched study with 12-month follow-up
Source: Front Cardiovasc Med. 2026 Jun 17;13:1808223. doi: 10.3389/fcvm.2026.1808223 (PMC13318561; doi:10.3389/fcvm.2026.1808223)

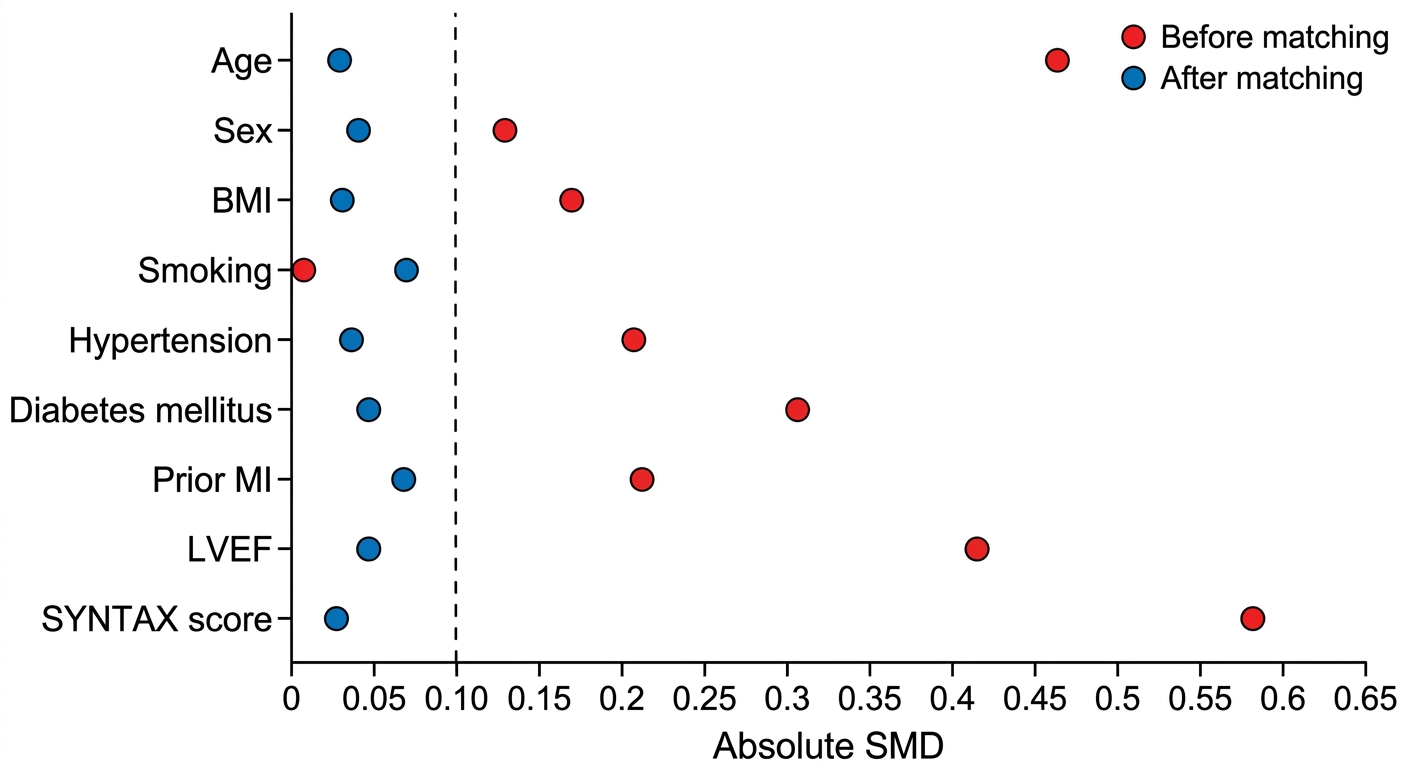

Supplement: Supplementary Figure S1 — Covariate balance before and after propensity score matching. The Love plot displays the absolute standardized mean differences for the prespecified covariates included in the propensity score model: age, sex, BMI, smoking status, hypertension, diabetes mellitus, prior myocardial infarction, left ventricular ejection fraction, and SYNTAX score. Red circles indicate the unmatched cohort and blue circles indicate the matched cohort. The dashed vertical line marks an absolute standardized mean difference of 0.10. After matching, all included covariates showed an absolute standardized mean difference <0.10. [file Image1.jpeg]

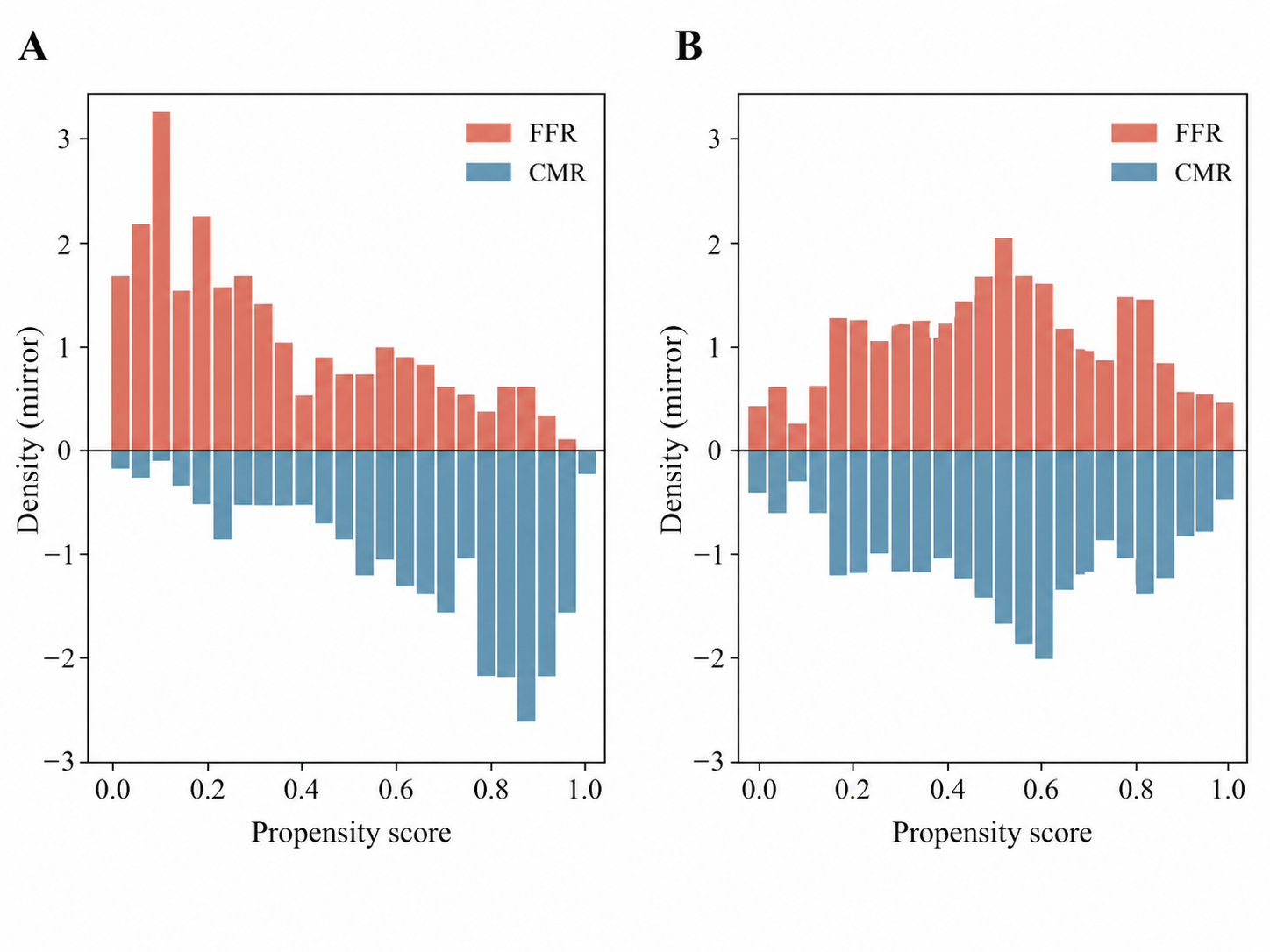

Supplement: Supplementary Figure S2 — Propensity score distributions before and after matching. (A) Distribution before matching. (B) Distribution after matching. The upper bars represent the FFR group, and the lower mirrored bars represent the CMR group. Propensity score overlap improved after matching, although some residual asymmetry remained at the upper tail of the distribution. [file Image2.jpeg]
